# Center-of-pressure total trajectory length is a complementary measure to maximum excursion to better differentiate multidirectional standing limits of stability between individuals with incomplete spinal cord injury and able-bodied individuals

**DOI:** 10.1186/1743-0003-11-8

**Published:** 2014-01-17

**Authors:** Jean-François Lemay, Dany H Gagnon, Sylvie Nadeau, Murielle Grangeon, Cindy Gauthier, Cyril Duclos

**Affiliations:** 1 School of Rehabilitation, Université de Montréal, Montreal, Canada; 2 Pathokinesiology Laboratory, Centre for Interdisciplinary Research in Rehabilitation of Greater Montreal, Institut de réadaptation Gingras-Lindsay-de-Montréal, 6300 avenue Darlington Montreal, QC H3S 2 J4, Canada

**Keywords:** Movement, Outcome assessment, Postural balance, Rehabilitation, Spinal cord injuries

## Abstract

**Background:**

Sensorimotor impairments secondary to a spinal cord injury affect standing postural balance. While quasi-static postural balance impairments have been documented, little information is known about dynamic postural balance in this population. The aim of this study was to quantify and characterize dynamic postural balance while standing among individuals with a spinal cord injury using the comfortable multidirectional limits of stability test and to explore its association with the quasi-static standing postural balance test.

**Methods:**

Sixteen individuals with an incomplete spinal cord injury and sixteen able-bodied individuals participated in this study. For the comfortable multidirectional limits of stability test, participants were instructed to lean as far as possible in 8 directions, separated by 45° while standing with each foot on a forceplate and real-time COP visual feedback provided. Measures computed using the center of pressure (COP), such as the absolute maximal distance reached (COP_max_) and the total length travelled by the COP to reach the maximal distance (COP_length_), were used to characterize performance in each direction. Quasi-static standing postural balance with eyes open was evaluated using time-domain measures of the COP. The difference between the groups and the association between the dynamic and quasi-static test were analyzed.

**Results:**

The COP_length_ of individuals with SCI was significantly greater (p ≤ 0.001) than that of able-bodied individuals in all tested directions except in the anterior and posterior directions (p ≤ 0.039), indicating an increased COP trajectory while progressing towards their maximal distance. The COP_max_ in the anterior direction was significantly smaller for individuals with SCI. Little association was found between the comfortable multidirectional limits of stability test and the quasi-static postural balance test (r ≥ −0.658).

**Conclusion:**

Standing dynamic postural balance performance in individuals with an incomplete spinal cord injury can be differentiated from that of able-bodied individuals with the comfortable limits of stability test. Performance among individuals with an incomplete spinal cord injury is characterized by lack of precision when reaching. The comfortable limits of stability test provides supplementary information and could serve as an adjunct to the quasi-static test when evaluating postural balance in an incomplete spinal cord injury population.

## Background

Following an incomplete spinal cord injury (SCI), most individuals will experience sensory and motor impairments at and below the level of the lesion. Due to the neural configuration of the spinal cord, an incomplete SCI will generally result in various degrees of lower extremity, trunk and upper extremity impairments depending on the level and the neural structures specifically affected by the lesion. These impairments often impact the ability to stand safely and execute functional activities in this position (e.g., multidirectional reaching). In fact, recent figures report a high incidence of falls (up to 75%) in individuals with an incomplete SCI, often resulting in physical injuries and decreased social participation [1,2]. Individuals with SCI identified loss of balance as one of the major factors contributing to falls [2]. Unfortunately, only a few studies have investigated how people with SCI control postural balance during quiet standing [3,4] and how it changes when the trunk, head and upper extremities are engaged in dynamic movements over a fixed base of support [4].

Postural balance could be defined as the ability to stabilize the body’s center of mass (COM) over its base of support (BOS) [5]. To preserve a state of balance, the COM position is constantly regulated by the position of the center of pressure (COP), defined as the point location of the ground reaction force vector on the surface of a force platform on which a person is standing [6]. Given the stabilizing role of the COP, numerous valid and reliable parameters [6,7] have been recommended to characterize performance during quasi-static stance (i.e., standing with no intended movement [8]. So far, evidence of quasi-static postural balance impairments in individuals with SCI has been demonstrated. For instance, these individuals are less stable when standing either with their eyes open or closed than able-bodied individuals [3]. Moreover, their use of visual information to maintain quasi-static stance is greater compared to healthy individuals [3]. However, a comprehensive assessment of postural balance should include quasi-static stance and dynamic activities since performance in both conditions does not usually correlate in healthy adults [9,10]; nevertheless, this issue remains controversial [11]. The signs of dynamic instability have been documented during walking in this population and have been mostly expressed by greater variability in the margin of stability, step length, step width and mediolateral and anteroposterior foot placement as compared to controls [12]. To quantify dynamic balance during functional activities, other comprehensive biomechanical models have also been proposed but have yet to be used in individuals with SCI [13-15].

Another method for assessing dynamic postural balance is by exploring the limits of stability, which can be defined as the maximum distance an individual is willing to move his/her centre of pressure (COP) in various directions without changing the configuration of the BOS and while remaining stable [16]. This usually involves the use of a forceplate that records COP displacement and a visual display that serves as feedback to maximize COP movement in specific directions. A multidirectional limits of stability test has recently been used to characterize dynamic sitting postural balance in individuals with SCI [17,18]. Individuals having no motor control over their abdominal and low back muscles had distinct ability on this test compared to those who had partial or full control, which tends to support its validity in a seated position [17]. However, standing imposes different biomechanical and dynamic constraints on stability compared to a sitting position, provided that, for example, the BOS is smaller and the COM higher. An analysis of the COP while standing in healthy young adults reveals that time-domain measures were larger and frequency-domain measures were smaller compared to sitting, an observation attributed to the body segment that is moving in each position [19]. The multidirectional limits of stability test thus requires further study before it can be recommended for evaluating postural balance while standing.

Therefore, we conducted a comparative study using a laboratory-based measure of dynamic postural balance, i.e., the comfortable multidirectional standing test, as well as a quasi-static postural stability test in both spinal cord injury and able-bodied individuals. The main purpose of this study was to quantify standing dynamic postural balance in individuals with SCI. The secondary purpose of this study was to measure the association of this test with quasi-static standing balance. We hypothesized that individuals with SCI would present a lower level of dynamic postural balance while standing as compared to able-bodied individuals. We also expected a low level of association with the quasi-static standing postural balance test.

## Methods

### Participants

Sixteen individuals with an incomplete traumatic SCI (American Spinal Cord Injury (ASIA) Impairment Scale = D) [20] and 16 able-bodied controls volunteered to participate in this study. Participants with SCI were recruited from both the inpatient and outpatient population of the SCI rehabilitation unit of the Institut de réadaptation Gingras-Lindsay-de-Montréal (IRGLM). All SCI participants could stand for 5 minutes without external support and walk independently for 10 m with or without a walking assistive device. Participants were excluded if they presented other concomitant neurological conditions in addition to the SCI or walking or balance difficulties prior to the SCI. None of the able-bodied participants reported having musculoskeletal or neurological impairments that would interfere with standing postural balance. Ethics approval was obtained from Research Ethics Committee of the Center for Interdisciplinary Research in Rehabilitation of Greater Montreal (CRIR-578-0111). Written consent was obtained after participants had read and understood the information about the research (Table 1).

**Table 1:** **Descriptive characteristics of the participants (n = 32), mean ( *SD *) and range**

	**SCI group (n = 16)**		**Able-bodies group (n = 16)**	
	**Mean ( *SD *)**	**Range**	**Mean ( *SD *)**	**Range**
Age (years)*	50.3 (17.4)	20–67	41.5 (13.2)	23–67
Height (cm)*	173.4 (6.5)	158–183	174.5 (6.4)	165–189
Mass (kg)*	79.6 (15.8)	53.6–105.5	83.4 (13.2)	65.8–123.5
Time post lesion (days)	318.3 (226.8)	15–740		
LEMS (/50) n = 14	45.3 (3.6)	39–50		
Natural speed (m/s)	1.02 (0.27)	0.53–1.39		

LEMS: lower extremity motor score.

*No significant difference between the groups.

### Clinical assessment

Demographic information pertaining to the date and type of accident, ASIA level and the presence of any relevant associated conditions were gathered from each subject’s chart. A physical therapist with 10 years of experience in the field of SCI rehabilitation conducted the lower extremity motor score assessment (LEMS) according to ASIA standards [20]. Natural walking speed was tested over a distance of 15 m. Participants were asked to walk at their usual walking speed without any walking assistive devices [21]. The middle 10 m section was timed using a stopwatch. The task was repeated three times and the resulting speeds were averaged.

### Laboratory assessment

All evaluations were conducted at the Pathokinesiology Laboratory of the Centre for Interdisciplinary Research in Rehabilitation of Greater Montreal (CRIR) located at the IRGLM. During the laboratory assessment, participants stood on two side-by-side forceplates embedded in the floor with their feet in a standardized position (heels 10 cm apart; feet abducted 20°) and their arms resting alongside their trunk.

#### Comfortable multidirectional limits of stability test

Following a familiarization period, participants were asked to lean from the starting position as far as possible at a comfortable self-selected speed in eight specific directions, each separated by 45° (anterior, right anterolateral, right, right posterolateral, posterior, left posterolateral, left, left anterolateral) and return to the initial position within a 15-second period. Participants were instructed to keep their arms alongside their trunk, to initiate the movement from the ankle instead of bending their trunk or their hips when leaning and to avoid raising their toes or heels [22]. These instructions were given to facilitate COP movement in the indicated direction within a standardized BOS and to limit compensatory movements. A flat screen placed 2 m in front of the participant displayed the real-time position of their COP as well as the boundaries within which they had to move. Each of the eight directions was tested twice for a total of 16 movements that were displayed in a random order determined by a computer. A research engineer coordinated the computerized data acquisition and storage at all times.

#### Quasi-static postural stability test

Participants were asked to stand still during two 45-second trials with their eyes open. Participants had to keep their feet in the same standardized position as described above [3].

### Data processing

Reaction forces were recorded for each task at a sampling frequency of 600 Hz. The resultant COP time series, computed from the tri-axial components of the combined reaction forces, was filtered with a fourth-order Butterworth zero-lag filter with a cut-off frequency of 5 Hz and then down-sampled (300 Hz) before analysis.

### Outcome measures

#### Comfortable multidirectional limits of stability test

Three main outcome measures were taken:

• COP_max_: represents the linear distance in mm between the initial and maximal positions of the COP in a given direction. The COP_max_ was calculated for each trial in the eight directions tested. Greater COP_max_ indicates better ability to move the COP in a specific direction.

• COP_length_: represents the total trajectory in mm followed by the COP from its initial position to its maximal position. The COP_length_ was also computed for each of the eight directions. Lower COP_length_ indicates a straighter movement when reaching in a specific direction, possibly indicating better precision of the COP movement.

• COP_area_: represents the area in mm^2^ defined by an ellipse fitting the COP_max_ in each of the 8 tested directions. Greater COP_area_ indicates better overall ability to move the COP in every direction.

The mean COP speed was also calculated for each trial by taking the total distance travelled between the COP starting and maximal (COP_max_) positions divided by the time taken to reach this distance. Each trial was then analyzed to be included in the final analysis. Data from trials where a loss of balance or a foot displacement occurred were also discarded. The angular error (representing the absolute difference in degrees between the targeted direction and the actual COP_max_ direction) was calculated for each trial. Only trials with an angular error ≤25° were included in the analysis to minimize the impact of imprecise COP directions. For each direction, the maximal value of the COP_max_ was taken as well as the corresponding COP_length_.

#### Quasi-static postural stability test

The mean values of the root mean square distance (RMS; mm), the mean COP velocity (MV; mm/s), and the COP sway area (SA; mm^2^/s) were computed for each trial from the COP time series [3,6,7]. Lower RMS, MV and SA values would indicate a better performance in terms of postural balance. The mean of the two trials was calculated for each parameter.

### Data analysis

Descriptive statistics (mean, standard deviation, range) were calculated for the participants’ characteristics and all laboratory outcome measures. Normality of the distribution was explored using the Shapiro-Wilk test. Among the comfortable multidirectional limits of stability test parameters, the COP_length_ in the right, right posterolateral, posterior and left directions of individuals with SCI, as well as the COP_length_ in the anterior and right posterolateral directions of the able-bodied participants were not normally distributed (p ≤ 0.032). The quasi-static postural stability test departed from normality in individuals with SCI for the MV (p ≤ 0.037). Non-parametric statistics were thus used when analyzing these data. Otherwise, all other data were normally distributed among the two groups and analyzed with parametric tests.

Independent t-tests were used to reveal differences in the demographic data of both groups of participants. For the comfortable multidirectional limits of stability test parameters, a two-way repeated measure analysis of variance (ANOVA) with one between factor (individuals with SCI and able-bodied individuals) and one within factor (eight tested directions) was conducted to detect the presence of significant differences. Since there was a significant interaction between the COP_max_ and the groups, independent t-tests or Mann–Whitney *U* tests were performed to reveal between-group differences for each direction, using a Bonferonni correction on the alpha value (p = 0.05/8 tested directions). Effect size were calculated for both the COP_max_ and the COP_length_ and were considered as being small (0.2 to 0.5), moderate (0.5-0.8) or large (>0.8) according to Cohen’s criteria [23]. Difference between the groups on the quasi-static test was quantified using independent t-test or Mann–Whitney *U* test according to the normality of the data. Further analysis on the COP_max_ where individuals with SCI were better than able-bodied controls was done by assessing the level of association with the COP_length_ in the same direction using Pearson’s product moment correlation coefficient. The between-group difference of the COP_area_ and the mean COP speed was assessed with an independent t-test.

The level of association between the comfortable multidirectional limits of stability test and the quasi-static postural stability test for individuals with SCI was explored using Pearson’s product moment correlation coefficients. Correlation coefficients confirmed good to excellent association when they were greater than 0.75 [24]. A statistical significance threshold was set at 0.05 for all tests unless otherwise specified. All statistical analyses were conducted using SPSS® software (version 20.0) (Chicago, IL).

## Results

### Between-group and within-group differences

For the comfortable multidirectional limits of stability test, a significant difference was found only in the anterior direction for the COP_max_ between the two groups of participants at a statistical significance level of 0.00625. However, individuals with SCI tended to have a greater COP_max_ when reaching laterally and posteriorly (12.1 to 17.5%). Able-bodied individuals had greater COP_max_ when reaching in anterior directions (9.3 to 29.5%).

**Figure 1:**
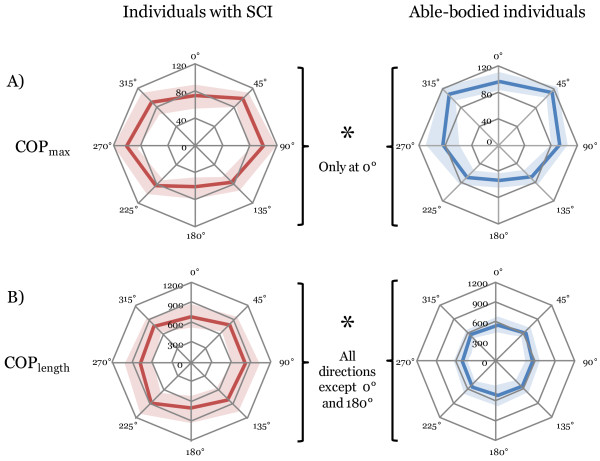
**Comparison of performance for two parameters of the comfortable multidirectional limits of stability test between individuals with a spinal cord injury and able-bodied individuals.** AL: anterolateral; PL: posterolateral. **A)** The COP_max_ represents the maximal distance reached by the center of pressure in every direction. Although differences in performance between the groups can be seen in most directions, none reached the adjusted level of statistical differences (0.00625 (0.05/8 directions)). **B)** The COP_length_ represents the length of the COP trajectory from the starting position to the maximal position in a given direction. Individuals with SCI had significantly longer COP_length_ in all but one direction (180°) as compared to able-bodied individuals.

**Figure 2:**
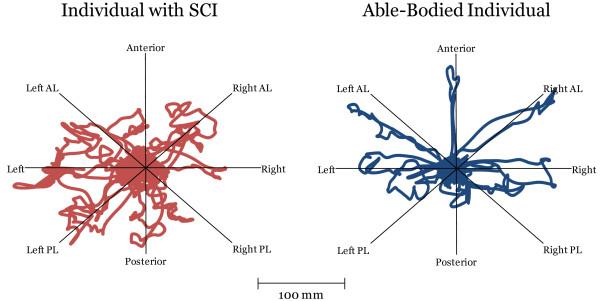
**An example of a COP displacement on the comfortable multidirectional limits of stability test performed by two individuals, one with and the other without SCI.** AL: anterolateral; PL: posterolateral. The individual with SCI displays less precision when reaching in all directions as compared to the able-bodied individuals, for whom most directions are easier to differentiate. Overall, reaching movements in the posterior direction tended to be smaller and less precise than in the anterior and lateral directions in both groups.

The COP_length_ for individuals with SCI was 22.2-35.8% greater than those of able-bodied individuals. This reached the significance level in all directions (p ≤ 0.001), except in the anterior and posterior direction (p ≤ 0.039). Both the COP_max_ and the COP_length_ tended to be smaller in the posterior directions as compared to anterior and lateral directions (Figures 1 and 2). Effect sizes were considered moderate to large.

There was a significant level of association between the COP_max_ and the COP_length_ in the posterior, left posterolateral and left directions for individuals with SCI (0.527 ≤ r ≤ 0.630, p ≤ 0.036).

The COP_area_ was not significantly different between SCI and able-bodied participants (p = 0.560) (Table 2). No difference was seen on the mean COP speed at the significance level of 0.00625. All COP parameters of the quasi-static postural stability test revealed reduced postural balance in individuals with SCI as compared to able-bodied participants (p ≤ 0.0002).

**Table 2:** Descriptive characteristics of the participants on the dynamic and quasi-static tests

**Test**	**Parameter**	**Specification**	**SCI group (n = 16)**	**Able-bodied group (n = 16)**	**Effect-size**	
			**Mean ( *SD *)**	**Mean ( *SD *)**	**( *d *)**	**(95% CI)**
Comfortable multidirectional limits of stability test	COP_max_ (mm)	Anterior*†	75.5 (15.6)	94.2 (13.7)	1.27	(0.48, 2.00)
		Right AL	100.6 (12.0)	110.0 (11.7)	0.79	(0.05,1.49)
		Right	101.8 (19.8)	89.0 (12.5)	0.77	(0.04, 1.47)
		Right PL	76.5 (17.6)	67.2 (14.8)	0.57	(−0.15, 1.26)
		Posterior	60.6 (18.2)	53.0 (10.9)	0.51	(−0.21, 1.20)
		Left PL	83.5 (17.9)	68.9 (14.7)	0.89	(0.14, 1.59)
		Left	102.6 (18.4)	90.0 (9.9)	0.85	(0.11, 1.55)
		Left AL	92.4 (19.3)	106.7 (14.2)	0.84	(0.10, 1.54)
	COP_length_ (mm)	Anterior	695.0 (191.5)	540.9 (121.7)	0.96	(0.21, 1.67)
		Right AL*	811.6 (171.5)	593.0 (99.6)	1.56	(0.73, 2.31)
		Right*	804.9 (225.4)	516.6 (107.6)	1.63	(0.80, 2.39)
		Right PL*†	765.6 (242.5)	516.3 (133.3)	1.27	(0.49, 2.00)
		Posterior	659.2 (220.9)	502.9 (173.2)	0.79	(0.05, 1.49)
		Left PL*	831.7 (221.7)	537.4 (161.4)	1.52	(0.70, 2.26)
		Left*	749.6 (228.7)	529.9 (123.6)	1.20	(0.42, 1.91)
		Left AL*	779.5 (156.8)	569.4 (87.3)	1.66	(0.82, 2.41)
	COP_area_ (mm^2^)		20181.8 (4527.8)	19332.4 (3557.1)	0.21	(−0.49, 0.90)
Quasi-static test	RMS (mm)	EO*	8.71 (2.67)	5.17 (1.63)	1.60	(0.77, 2.35)
	MV (mm/s)	EO*†	16.00 (6.20)	7.52 (2.00)	1.84	(0.97, 2.62)
	SA (mm^2^/s)	EO*†	40.41 (29.00)	8.98 (4.75)	1.51	(0.69, 2.26)

*Significant between-group difference (Independent t-test or Mann–Whitney *U* test (†); p < 0.05). COP_max_: maximal distance reached by the COP in the indicated direction; COP_area_: surface of the octagon encompassing the eight COP_max_ directions of an individual. COP_length_: length of the COP trajectory from the starting position to the maximal position of the COP; AL: anterolateral; PL: posterolateral; RMS: root mean square distance of the COP; MV: mean velocity of the COP; SA: Sway-area of the COP; EO: eyes open; *d*: Cohen’s *d*.

### Correlation between the comfortable multidirectional limits of stability test and the quasi-static postural stability test for individuals with SCI

**Figure 3:**
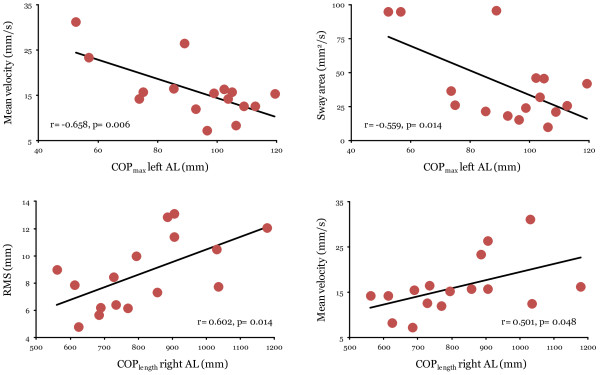
**Scatter plots representing statistically significant associations between the comfortable multidirectional limits of stability test and the quasi-static postural steadiness test for individuals with SCI.** AL: anterolateral, RMS: Root mean square distance. Although the four relationships displayed are significant, they have to be interpreted with caution since all the other relationships between parameters of the quasi-static postural steadiness test and the comfortable multidirectional limits of stability test were not significant for individuals with SCI. Moreover, there seems to be a slight ceiling effect in terms of the sway area parameter in the quasi-static postural steadiness test seems on the second scatter plot.

For individuals with SCI, there was a limited number of significant associations (4 out of 48 possible associations) between the parameters of the comfortable multidirectional limits of stability test and the quasi-static postural stability test (Table 3). A moderate level of association was present between the COP_max_ in a left anterolateral direction and the MV and SA parameter of the quasi-static postural stability test (−0.658 ≤ r ≤ −0.559; p ≤ 0.014), as well as between the COP_length_ in the right anterolateral direction and the RMS and MV parameters (0.501 ≤ r ≤ 0.602; p ≤ 0.048) (Figure 3).

**Table 3:** Correlation matrix between the dynamic and the quasi-static test for individuals with SCI

**Quasi-static test**	**Comfortable multidirectional limits of stability test**							
	**COP**_**max**_							
	**Anterior**	**Right AL**	**Right**	**Right PL**	**Posterior**	**Left PL**	**Left**	**Left AL**
RMS†	−0.194	0.217	−0.006	0.040	0.468	0.475	0.325	−0.275
MV†	−0.352	0.277	−0.232	−0.393	−0.080	0.361	0.064	−0.658**
SA†	−0.387	0.142	−0.204	−0.239	0.177	0.355	0.167	−0.559*
	**COP**_**length**_							
	**Anterior**	**Right AL**	**Right**	**Right PL**	**Posterior**	**Left PL**	**Left**	**Left AL**
RMS†	−0.067	0.602*	0.365	0.004	0.441	0.138	0.241	0.366
MV†	0.050	0.501*	0.173	−0.133	0.290	0.190	0.091	0.081
SA†	0.011	0.482	0.120	−0.047	0.292	0.149	0.135	0.143

†Pearson’s product moment correlation coefficient. *: p ≤ 0.05, **: p ≤ 0.01.

RMS: root mean square distance of the COP; MV: mean velocity of the COP; SA: sway area of the COP; COP_max:_ maximal distance reached by the centre of pressure in the indicated direction. COP_length_: length of the COP trajectory from the starting position to the maximal position of the COP; AL: anterolateral; PL: posterolateral.

## Discussion

In this study, we have found that a task testing the limits of stability in SCI individuals who are community ambulators, is mainly characterized by an increase in the distance travelled by the COP when going to its maximal position rather than by a limitation in the absolute distance reached by the COP in the intended direction. Therefore, using COP_length_ may better characterize performance of individuals with SCI. When the COP_max_ is greater in individuals with SCI as compared to able-bodied individuals in some direction, it is associated with the COP_length_, which further support the use of this measure in a SCI population. To our knowledge, this is the first study to report such a finding in this population. In accordance with our second hypothesis, we also found little association between the quasi-static and the dynamic balance parameters.

### Performance of individuals with SCI versus able-bodied individuals on the comfortable limits of stability test

COP_length_ is calculated from the total excursion of the COP on its way toward its maximal position in an indicated direction. COP_length_ of individuals with SCI was greater than that of able-bodied individuals in all directions. This increased COP excursion could be interpreted as a decrease in movement precision: whereas COP movement of able-bodied individuals tended to move in a straighter manner, individuals with SCI displayed more extraneous movements on their way to their maximal position, increasing the COP_length_. Similar results have been found in other populations presenting neurological deficits such as Parkinson’s disease. Ondo et al. found that individuals with Parkinson’s disease had a significantly increased path sway on the Smart Balance Master limits of stability test as compared to able-bodied individuals, a test comparable to the one performed in our study [25].

Many factors may be responsible for this decrease in movement precision during the comfortable multidirectional limits of stability test in individuals with SCI. Since a SCI generally alters the integrity of the various sensory tracts travelling within the spinal cord, the somatosensory contribution to postural balance may therefore be decreased, which in turn, could explain the less precise movement of the COP. Clinical evaluation of sensory function revealed residual sensory deficits in most of our participants with SCI. In fact, a varying degree of foot anesthesia induced from various techniques causes a proportional increase in COP motion while standing [26,27]. Moreover, an increase in visual contribution to postural balance in people with SCI as compared to able-bodied controls does exist [3]. This may represent a compensatory mechanism for a decreased sensory perception in the lower extremity. Future studies could identify to what extent sensory deficits following SCI is a major contributor to observed standing postural balance deficits.

Lower-extremity muscle function is another factor influencing control of standing postural balance in various populations [28-31]. Although our participants with SCI had sufficient lower extremity strength to assume a standing position, a certain deficit in strength was present in the ankle dorsiflexors and plantarflexors, as indicated by the LEMS evaluation. It is known that the location of lower-extremity muscle strength influences balance capability differently. For example, Horlings et al. demonstrated that distal muscle weakness more significantly influences postural stability than proximal muscle weakness [28]. Future studies using a dynamometric evaluation of lower extremities could help identify those muscle groups associated with increased COP_length_ in each direction.

Contrary to our initial hypothesis, individuals with SCI had similar COP_max_ compared to able-bodied controls as indicated by the lack of significant differences in all but one direction and on the overall measure given by the COP_area_. Interestingly, these findings correspond to those of Gauthier et al. who showed that SCI individuals who had partial or full control over their abdominal and lower trunk muscles could bring their COP to a similar distance from their base of support to that of able-bodied individuals when performing a similar postural balance test while sitting [17]. This may seem surprising considering that standing is inherently less stable than sitting [19,32] and could thus lead to greater differences in performance between impaired and normally functioning individuals. However in our study, individuals with SCI had adequate motor recovery in their trunk muscles to be able to assume a standing position and lean in various directions. This may in part explain the lack of difference seen in COP_max_. Yet the significant groups vs. directions interaction indicates that individuals with SCI and able-bodied participants differed on how they performed in various directions. More specifically, individuals with SCI had greater COP_max_ in lateral directions while able-bodied individuals had greater COP_max_ in anterior and antero-lateral directions. Complementary studies including a larger sample of participants could help to confirm whether or not a difference in COP_max_ between the groups exists. Greater COP_max_ in some directions cannot be attributed to different foot placements since foot placements were standardized using a template. This is also supported by the absence of significant difference between the groups on the COP_max_ in a posterior, right and left directions.

Ankle plantarflexor muscle groups are known to influence anteroposterior COP excursions [31] and are especially activated when controlling anterior body displacement with respect to the base of support [33]. Since most of our individuals with SCI had residual distal lower-extremity weakness, we could hypothesize that the lower COP_max_ in the anterior and anterolateral directions may be explained by this lack of strength. Individuals with SCI could therefore limit anterior COP displacement in order to take into account their decreased ability to control the COP using their ankle plantarflexors when reaching the limits of stability in this direction.

On the other hand, individuals with SCI had greater COP_max_ in the lateral directions than able-bodied individuals. Body displacements in lateral directions are under the control of hip abductor muscle groups [33], which did not achieve full recovery in our group of individuals with SCI. A possible explanation for this better performance is that those individuals with SCI who outperformed able-bodied individuals in these directions did it at the expense of precision, as indicated by the COP_length_. This hypothesis is partly supported by the significant positive correlation found between the COP_max_ and the COP_length_ in the posterior, left and left posterolateral directions where individuals with SCI were found to outperform able-bodied individuals. This indicates that, in these directions, individuals with SCI who could displace their COP further were generally less precise than those presenting more limited COP displacements. Since the comfortable multidirectional limits of stability test challenges the postural control system and attentional resources, some individuals may have favored moving less precisely in the displayed direction in order to achieve a maximal performance on the COP_max_. Therefore, repeating this test while imposing no constraint on the movement precision may have yielded different COP_max_ results.

### Association between quasi-static and dynamic postural balance tests

This study also yields little association between dynamic and quasi-static postural balance for individuals with SCI. As mentioned earlier, only four of the 48 possible combinations were found to be statistically significant. This limits the inferences that can be drawn from these associations. These results support a previous study reporting a lack of significant correlation between the static eyes open test and the limits of stability test of the Smart Balance Master, the later sharing some similarities with the comfortable limits of stability test [4]. This is also in line with most studies exploring the association between measures of static and dynamic balance among individuals with stroke or able-bodied individuals [9,10,34]. In spite of these remarks, parameters of the COP during quasi-static stance correlated with the comfortable multidirectional limits of stability test in only two directions (left and right anterolateral directions). Further research including more participants with a wider range of balance deficits would be necessary to determine the significance of this specific result.

### Study limitations

Our study suffers from a few limitations needing consideration when interpreting these results. Firstly, all our participants with SCI were community ambulators. Many participants with SCI had near normal walking ability, as indicated by the mean natural speed of 1.02 m/s, which is close to the 1.06 m/s value required to be considered as a safe community ambulator [35]. It is thus possible that our group of SCI participants was not representative of an actual population of individuals with traumatic incomplete SCI. These results are therefore not applicable to those individuals who are starting to assume a standing position. This could have limited the possibility to find differences between our two groups. However, although statistical power of the study was in part limited by the small number of participants in each group, effect sizes were at least moderate for the COP_length_ in the comfortable multidirectional limits of stability test. Therefore, this statistic supports the fact that our sample size was sufficient to find differences between the groups.

As is often done in other studies, COP-based measures were not normalized using the dimension of the base of support or foot length [17,18]. However, our two groups did not differ in height. Since foot position was standardized and monitored during the study, we therefore presume that this normalization would not have changed our main conclusions. Although participants were told to initiate the movement from the ankle instead of bending their trunk or their hips when leaning, some degree of trunk and hip compensation of varying degrees was present among participants and directions. We thus suspect that the actual performance may be partially related to the strategies used. Adding a kinematic analysis to our protocol may have helped to identify biomechanical markers associated with the difference in performance seen among both groups.

It is possible that the performance of individuals with SCI is potentiated because of the visual feedback provided during this test. In fact, Sayenko et al. have shown that visual feedback can improve standing balance performance in individuals with incomplete SCI [36]. A test performed without visual feedback could have been more reflective of balance capabilities of each participant and could have generated more differences between the groups. Normal dynamic postural balance activities such as walking occur without on-screen visual feedback on actual performance. Therefore, how the results from this study can be generalized to other dynamic balance activities remains to be explored.

Although 15 seconds were given to maximally displace the COP in the indicated direction and come back to the initial position, no actual control on the speed of movement was given. However no difference was seen between the groups in the mean COP speed. Thus this factor could not explain the differences in performance (i.e., COP_length_) seen between the groups. We did not analyze the return from the maximal position to the initial position, which may have provided further insight into dynamic balance performance in individuals with SCI in the comfortable limits of stability test. Reliability and minimal detectable change of the COP_max_ and the COP_length_ were not assessed. Since the standard error of measurement is unknown for these parameters, this limits the inferences that can be drawn from differences in performance seen between the groups of participants. Lastly, we did not apply a correction for multiple comparisons (e.g., Bonferonni’s) to the correlational analysis. For this reason, the correlation that were significant must be interpreted with caution since a possibility of a type I error exists.

## Conclusion

The comfortable multidirectional limits of stability test can characterize dynamic postural balance in individuals with SCI. More precisely, a measure of movement precision (i.e., COP_max_) could differentiate the performance of SCI individuals from that of able-bodied controls. Although balance during quasi-static standing is impaired in individuals with SCI, no definitive association was found between this evaluation and the dynamic test under investigation. A comprehensive evaluation of postural balance should therefore include items assessing both its static and dynamic components.

## Abbreviations

SCI: Spinal cord injury; COM: Center of mass; BOS: Base of support; COP: Center of pressure; AIS: ASIA impairment scale; IRGLM: Institut de réadaptation Gingras-Lindsay de Montréal; LEMS: Lower extremity motor score; CRIR: Centre for Interdisciplinary Research in Rehabilitation of Greater Montreal; RMS: Root mean square distance; MV: Mean COP velocity; SA: COP sway area; ANOVA: Analysis of variance.

## Competing interests

The authors declare that they have no competing interests.

## Authors’ contributions

JFL: Conception and design, acquisition, analysis and interpretation of data, drafted the article. DG: Conception and design, analysis and interpretation of data, drafted the article. SN: Conception and design, analysis and interpretation of data, drafted the article. MG: Conception and design, acquisition of data. CG: Conception and design, acquisition of data. CD: Conception and design, acquisition of data. All authors read and approved the final manuscript.

## Authors’ information

School of Rehabilitation (http://www.readap.umontreal.ca), Université de Montréal, Montreal, Canada.

Pathokinesiology Laboratory (http://www.pathokin.ca), Centre for Interdisciplinary Research in Rehabilitation of Greater Montreal — Institut de réadaptation Gingras-Lindsay de Montréal, Montreal, Canada.
